# Supplementary material for: Tumor Suppressor miRNA-503 Inhibits Cell Invasion in Head and Neck Cancer through the Wnt Signaling Pathway via the WNT3A/MMP Molecular Axis
Source: Int J Mol Sci. 2022 Dec 14;23(24):15900. doi: 10.3390/ijms232415900 (PMC9786678; doi:10.3390/ijms232415900)

## Slide 1
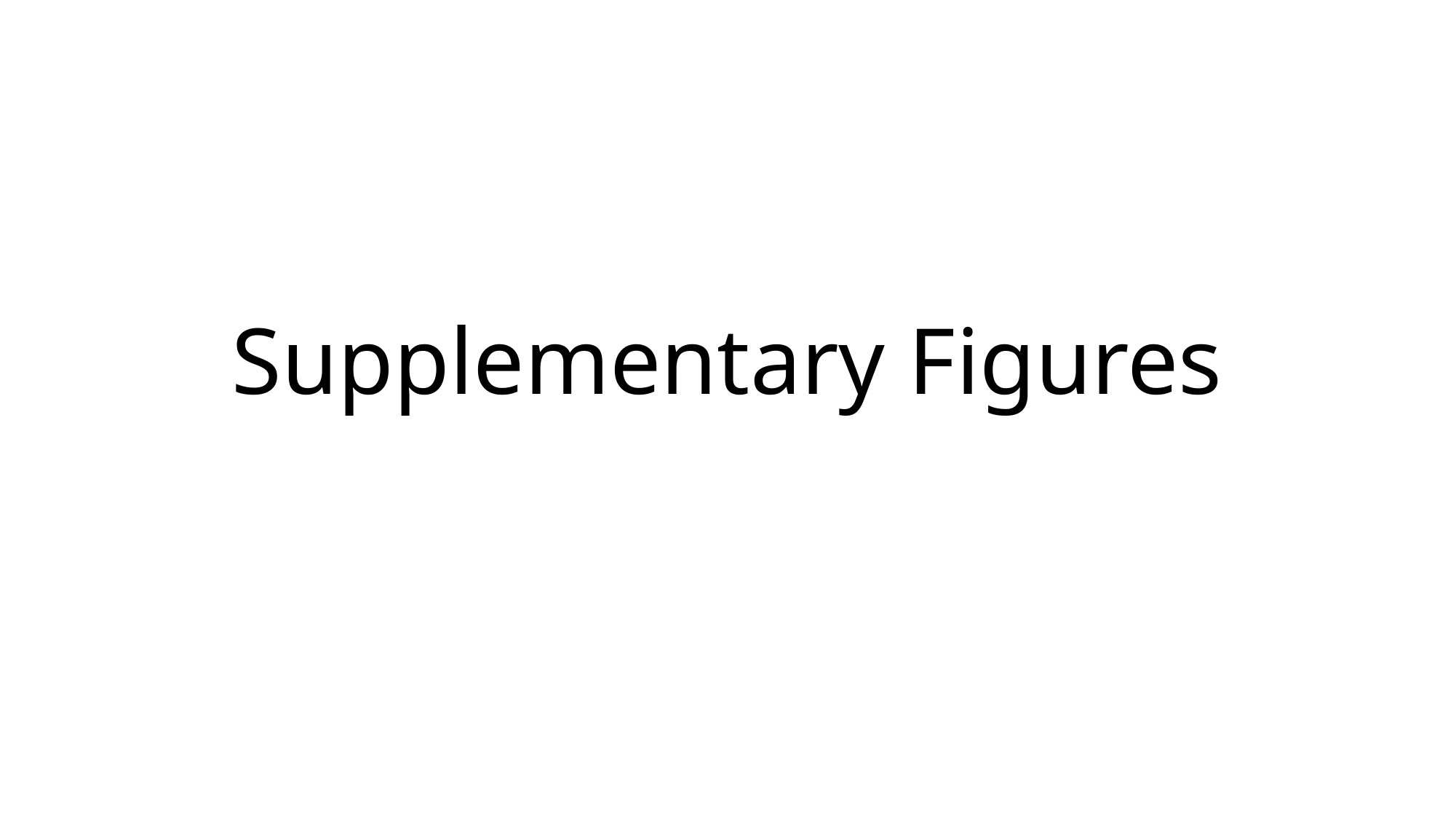

# Supplementary Figures

## Slide 2
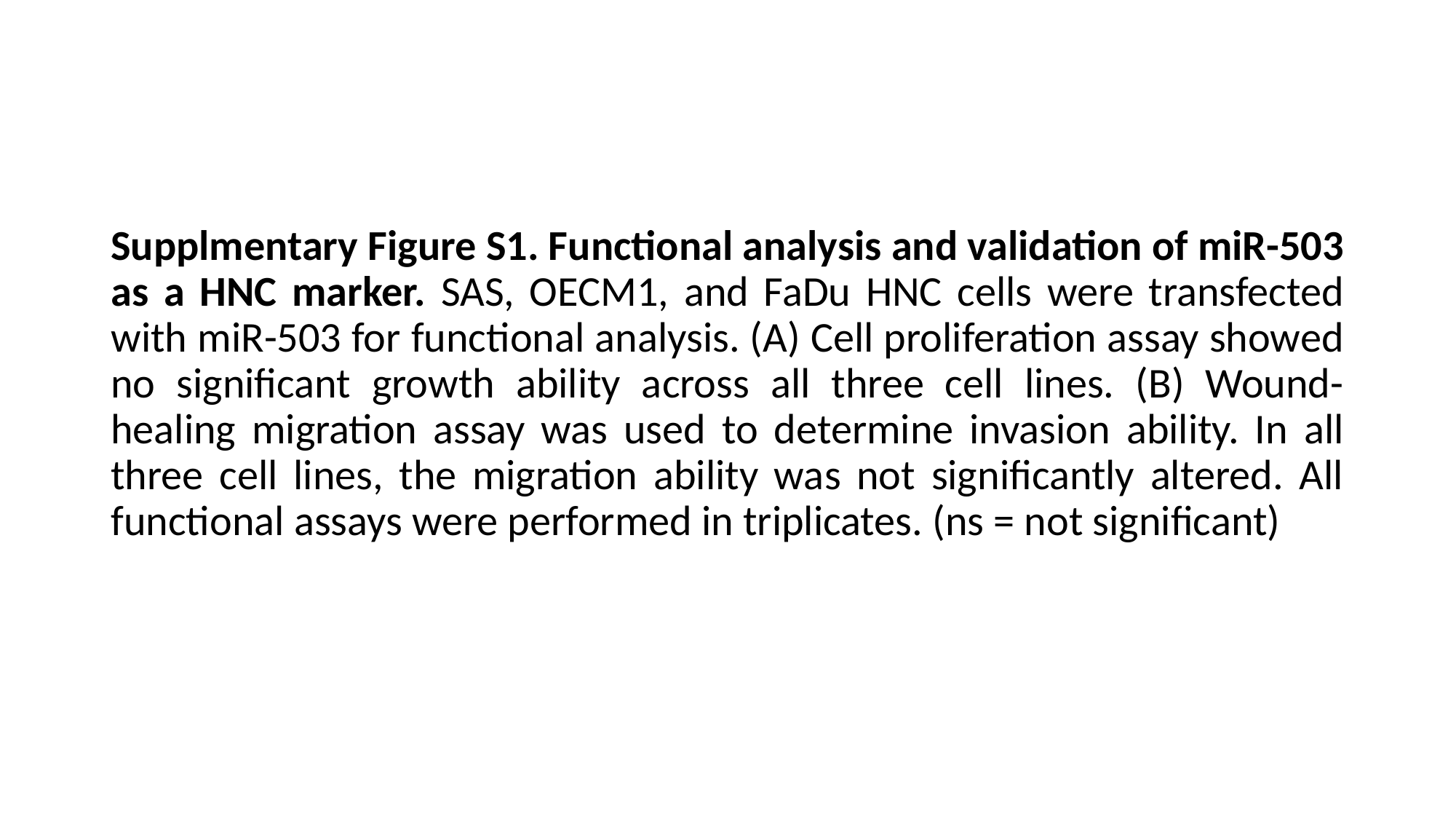

Supplmentary Figure S1. Functional analysis and validation of miR-503 as a HNC marker. SAS, OECM1, and FaDu HNC cells were transfected with miR-503 for functional analysis. (A) Cell proliferation assay showed no significant growth ability across all three cell lines. (B) Wound-healing migration assay was used to determine invasion ability. In all three cell lines, the migration ability was not significantly altered. All functional assays were performed in triplicates. (ns = not significant)

## Slide 3
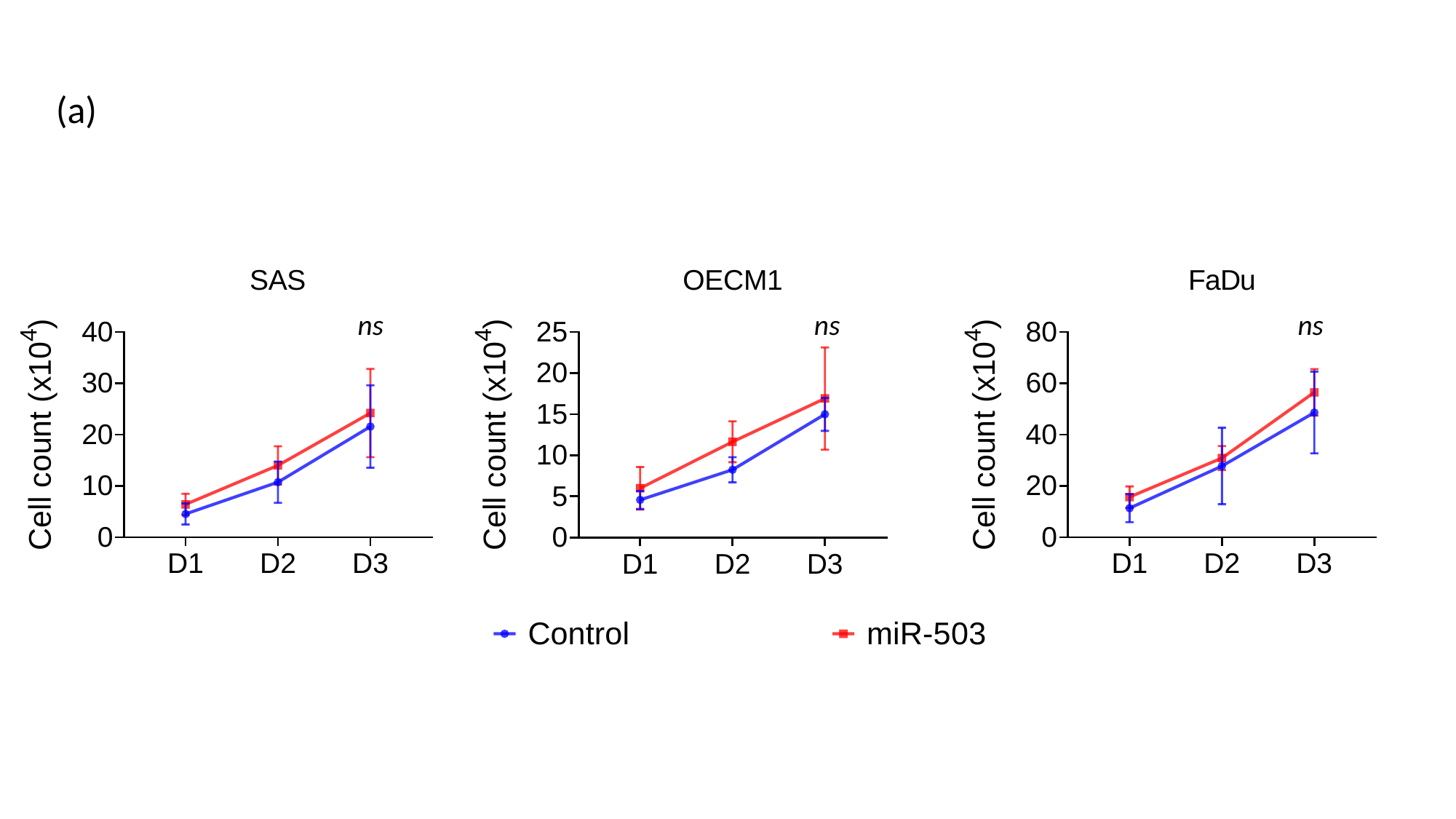

(a)

## Slide 4
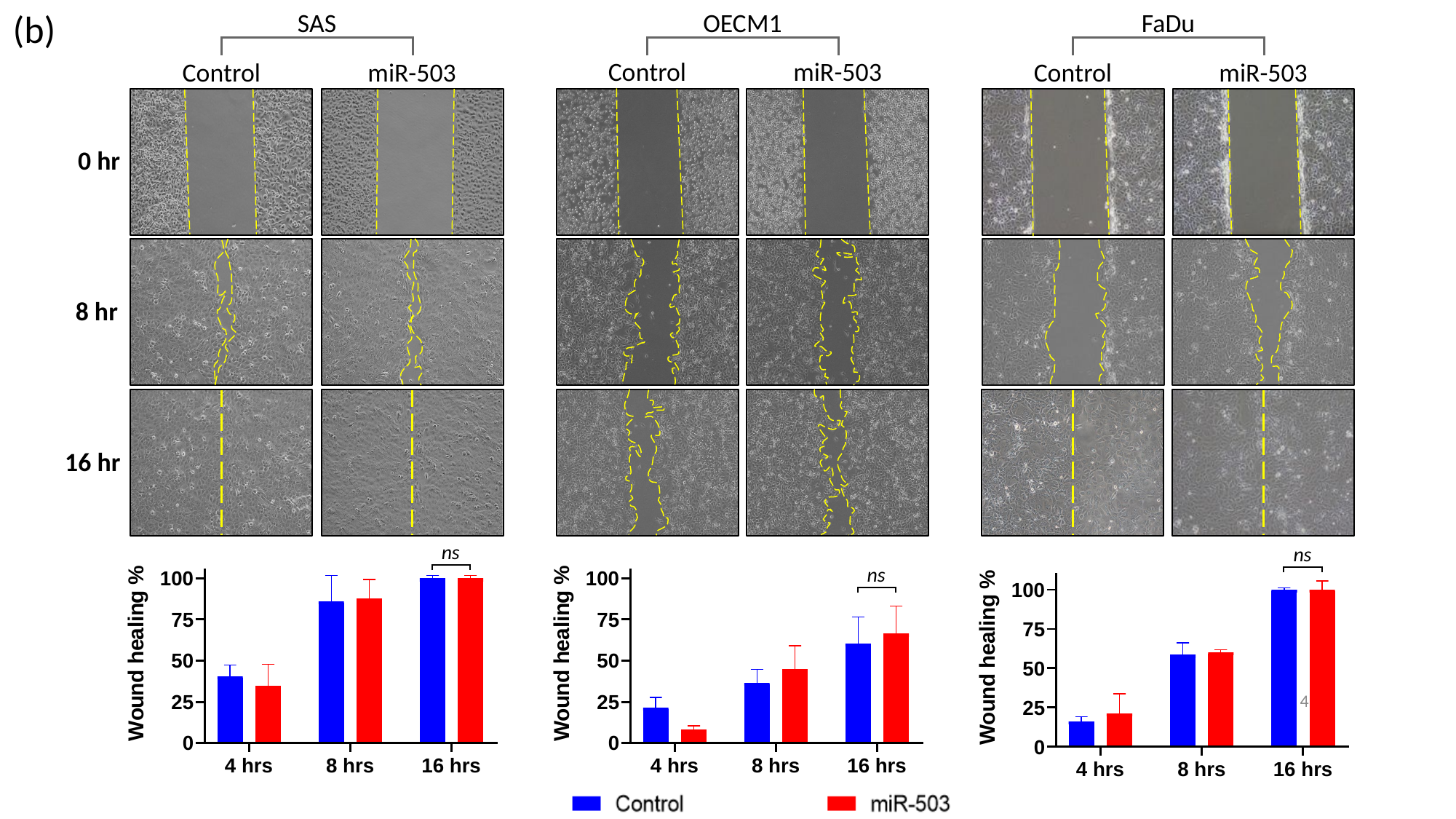

(b)

## Slide 5
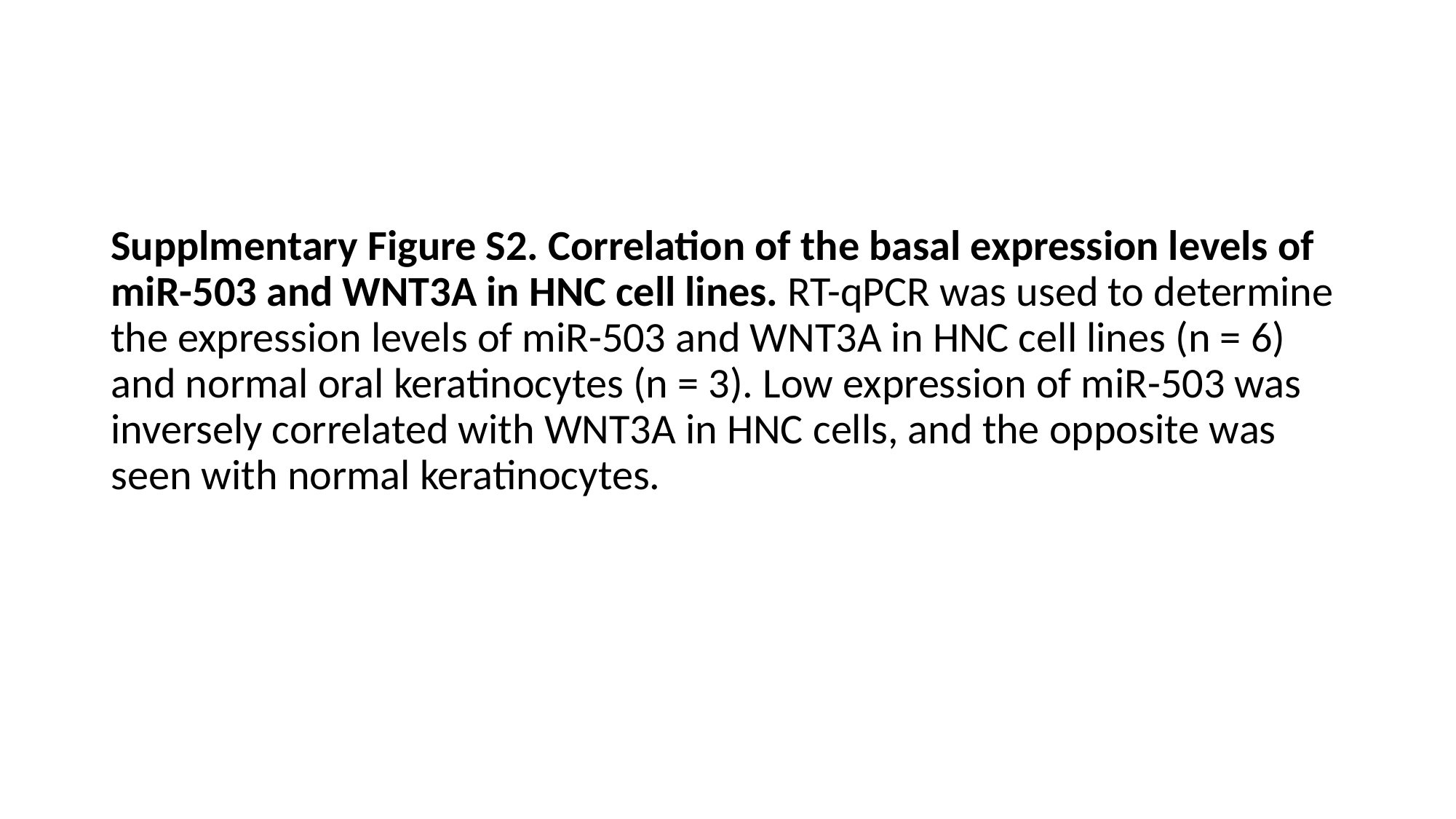

Supplmentary Figure S2. Correlation of the basal expression levels of miR-503 and WNT3A in HNC cell lines. RT-qPCR was used to determine the expression levels of miR-503 and WNT3A in HNC cell lines (n = 6) and normal oral keratinocytes (n = 3). Low expression of miR-503 was inversely correlated with WNT3A in HNC cells, and the opposite was seen with normal keratinocytes.

## Slide 6
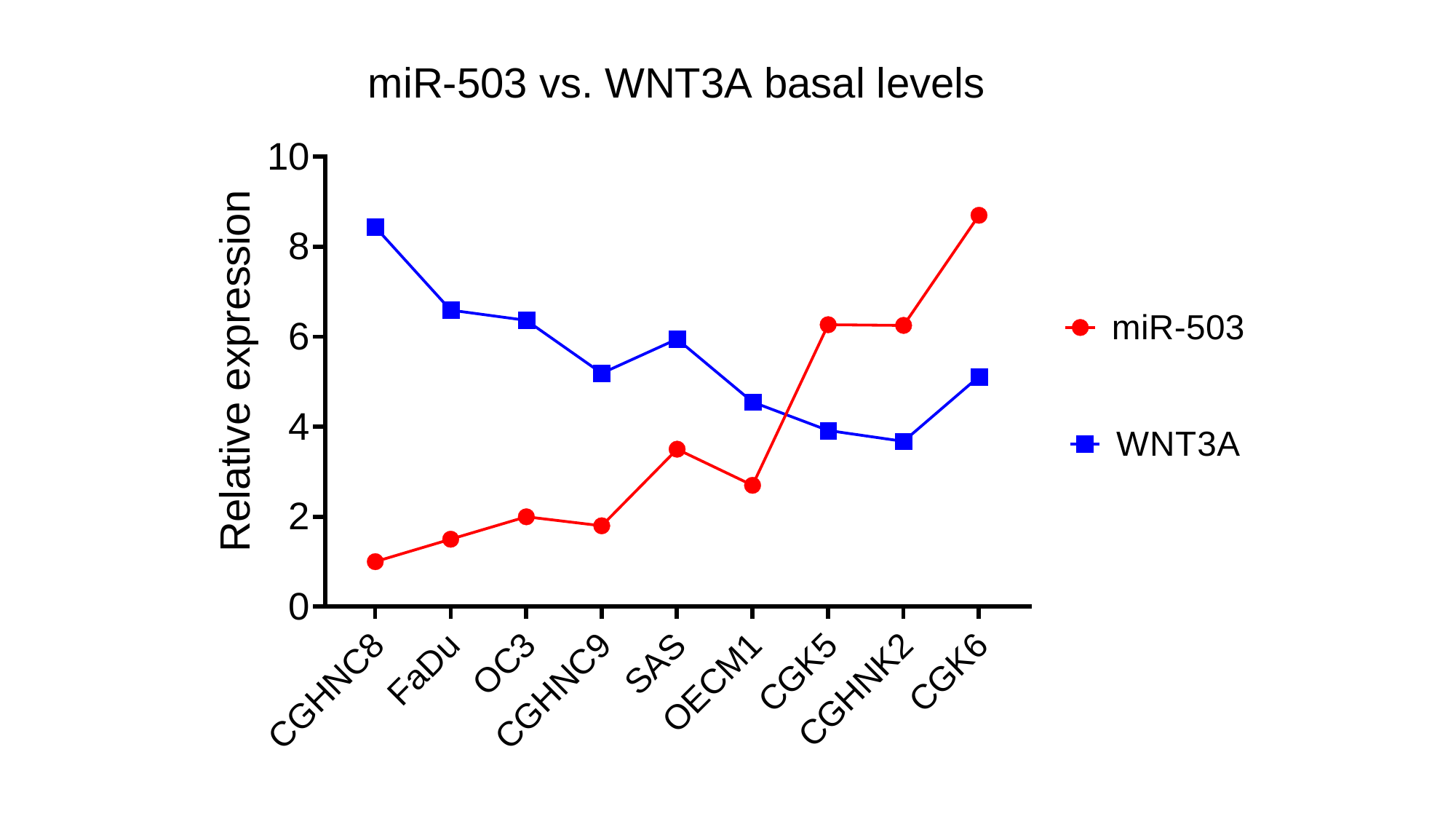

Supplement: Supplementary file 1 [file ijms-23-15900-s001.zip › Supplementary Figures.pptx]
